# Typology of Iranian farmers' vulnerability to the COVID-19 outbreak

**DOI:** 10.3389/fpubh.2022.1018406

**Published:** 2022-12-22

**Authors:** Somayeh Moradhaseli, Pouria Ataei, Hamid Karimi, Sara Hajialiani

**Affiliations:** ^1^Department of Agricultural Extension and Education, Faculty of Agriculture, Tarbiat Modares University, Tehran, Iran; ^2^Department of Agricultural Extension and Education, Faculty of Agriculture, University of Zabol, Zabol, Iran; ^3^Department of Agricultural Extension and Education, Faculty of Agriculture, Razi University, Kermanshah, Iran

**Keywords:** agricultural development, agricultural extension and education agents, COVID-19, farmers' vulnerability, farmers health

## Abstract

**Context:**

Presently, farmers are faced with a new crisis caused by the outbreak of COVID-19. On the one hand, they are vulnerable to such respiratory diseases due to the nature of their farming activity. On the other hand, they will definitely be influenced by the pandemic in different aspects no matter if they do not contract the infection. So, this research aimed to study the vulnerability of farmers to the COVID-19 pandemic.

**Methods:**

The present study was conducted using the quantitative approach and a descriptive-survey methodology. The statistical population was composed of farmers in Kermanshah province, Iran (*N* = 126,900). The sample (*n* = 382) was taken by the multistage stratified sampling technique with proportional allocation. The research instrument was a self-designed questionnaire whose face and content validity was confirmed by a panel of relevant experts and its reliability was supported in a pilot test.

**Results:**

The main damages of the pandemic to the farmers were found to be the increased costs of production, permanent or seasonal unemployment, reduced access to crop sale markets, and reduced control over pests and diseases at farms. The results revealed that the means of environmental, agronomic-vocational, and economic vulnerability were greater than the scale mean. The results also illustrated significant differences in the means of economic, psychological-social, agronomic-vocational, and environmental dimensions of vulnerability. Among these dimensions, the variable of agronomic-vocational vulnerability had the highest mean, and the variable of psychological-social vulnerability had the lowest mean.

**Conclusion:**

Farmers have been one of the groups most severely influenced and damaged by the pandemic in various aspects. In this regard, organizations and institutions in charge of different agriculture sections, especially the Office of Agricultural Extension and Education, must develop practical strategies to reduce the effect of the pandemic on the agricultural sector. Identifying the dimensions and parameters of farmers' vulnerability in the face of COVID-19 can provide new and appropriate solutions to relevant planners and policymakers.

## Introduction

The COVID-19 pandemic has influenced all aspects of human life, including socio-cultural, economic, and environmental dimensions, and human interactions the most. The outbreak of the infection has raised the fear of economic crisis and recession. Social distancing and restrictions on commuting have reduced the workforce in the economic sectors and resulted in the loss of many jobs (1). It is estimated that COVID-19 has destroyed billions of dollars of income in the global economy. In this regard, rural people, nomads, and farmers have been less considered in calculations. In addition, agriculture, villages, and nomads are mostly underestimated in these service-based calculations (2) whereas about 1.3 billion workers are estimated to be employed in the agricultural sector for a wide range of products, which accounts for half of the total workforce of the world (3). However, even though many people work in this sector and it is considered an important economic sector in most countries, agriculture is a high-risk industry with a high rate of injuries (4–6) and the highest levels of risk indicators (7–9).

Presently, farmers are faced with a new crisis created by the COVID-19 pandemic. On the one hand, respiratory diseases are common among farmers due to the nature of their work and their encounter with harmful biological factors in the workspace, such as viruses, fungi, and parasites, and their contact with the dust created by cereals, legumes, and pests and various chemical pesticides and fertilizers (10–13) so that farmers are already in the disease-vulnerable group of professions. On the other hand, farmers may not contract the infection, but they will surely be influenced by its outbreak (14).

According to FAO and WHO, farmers are a community that has severely been injured by the COVID-19 pandemic. As the main suppliers of the food chain, they are currently struggling with many production, livelihood, and social-mental challenges. In the meantime, they should keep farming to ensure not only their own livelihood but also the supply of national and international food, and in turn, food security (14). In this respect, research shows that farmers and producers in Europe are faced with growing problems and pressures due to the COVID-19 pandemic (15). In the US too, the pandemic has not only created a new type of crisis for the agricultural sector but it has also put American farmers in a tough situation (16). Iranian farmers have also been injured by the pandemic (17, 18). The pandemic has aggravated the vulnerability of the agricultural sector in Iran, which is already suffering from fundamental issues and challenges, e.g., lack of investment, low productivity, and market and production inefficiency (19).

This reflects the extreme vulnerability of farmers in this specific time period. Vulnerability has been defined by researchers in various professional settings, which varies with the goals and methods used. These differences make it difficult to have a universally accepted definition of vulnerability (20). Presently, there are over 25 different definitions and methods for vulnerability (21). Vulnerability is a situation in which the family loses the ability to cope with adverse conditions and falls into a situation in which it faces food, job, social, and health insecurity (22). Vulnerability is a multidimensional concept that varies with temporal and spatial scales and depends on economic, social, geographic, demographic, cultural, institutional, governmental, and environmental factors (23). However, the assessment of vulnerability provides a framework for identifying the social, economic, and environmental reasons for a disaster (24).

As was noted, farmers are exposed to numerous injuries due to the nature of the farming profession—injuries that either threaten their health, e.g., hazards, disease outbreaks, and work incidents (25, 26) or injuries that threaten their families' food security and income, e.g., natural disasters and market shocks (27, 28). Addressing these issues for finding solutions to reduce agricultural vulnerability requires a comprehensive and integrated management program with special attention to the risk of vulnerability and resilience of the target population (29, 30). Various studies have dealt with farmers' vulnerability in different fields. Ashtab and Sharifzadeh studied the social, economic, and environmental dimensions of farmers' livelihood vulnerability to drought. The results revealed that farmers were more vulnerable to water resources (among environmental measures) and social networks (among social measures) (31). Jamshidi et al. addressed farmers' vulnerability caused by climate change. According to their results, smallholders are more vulnerable to climate change (32). Measures like training, income, and access to infrastructure and credits can greatly help to mitigate vulnerability (33). Farmers' vulnerability to climate change has been found to be significantly affected by training, credits, membership in established organizations, unemployed family members, non-farming revenue, and environmental and drought warnings (34). Erikson et al. state that economic, social, political, and local factors affect vulnerability components at the farmer household level (35).

However, research about vulnerability to the COVID-19 outbreak showed that the COVID-19 pandemic injured farmers in different aspects as they failed to sell their crops and lost the market (1). With the closure of local markets for the sake of alleviating the risk of contagion, smallholders have had no way to sell their commodities (36). On the other hand, since farming highly depends on seasonal labor, it has faced labor shortages due to travel restrictions, which has reduced its production level. Furthermore, farmers' access to inputs and other production resources has been affected by the pandemic-related restrictions. Bochtis et al. concluded that shutdown reduced labor availability for important agricultural practices, such as vegetable cultivation, fruit picking, and so on (4, 37). According to FAO, COVID-19 has influenced agriculture in two important aspects—food supply and demand. These two aspects are directly related to food security, so food security has been jeopardized (14). Economic, psychological, and human aspects of vulnerability had the highest effect on food insecurity during the initial COVID-19 lockdown (38).

Also, FAO emphasizes that farmers will be challenged by limited access to markets to sell their products or buy inputs or by the increased prices of foodstuff and limited purchasing power in the future (39). Brewin concludes that most destructive secondary implications of the COVID-19 pandemic for the sustainability of agricultural systems are relevant to the whole world. These implications are related to, for example, excessive decrease in demand for food trade services, labor supply, transportation capacity, and food and crop production, which are influential on the reduction of farmers' production (40). Iese et al. studied the effects of the COVID-19 outbreak and enumerated some consequences as the reduction of agricultural production, the loss of food availability and revenue due to the limited access to local markets and the loss of international markets, the increase in social conflicts such as land conflicts, valuable crop and animal stealing, environmental degradation due to the immigration from rural to urban areas, and the decreased availability of seedlings, seeds, equipment, and labor (41). On the other hand, research has shown that pandemics influence people's mental health (42, 43).

Given the abovementioned points, it should be considered that when an issue spreads throughout the world so extensively, its effects cannot be expected to eliminate after a while and we should be ready for its long-lasting effects in different aspects to be able to help farmers in critical conditions. On the other hand, as with other crises those farmers have faced, their vulnerability to the COVID-19 pandemic can be scrutinized from different aspects. The present research tries to answer the question as to how the COVID-19 pandemic has injured farmers. Meanwhile, Kermanshah province is considered one of Iran's agricultural hubs and is privileged in terms of land and suitable climate. The province is considered one of the three centers of wheat production in Iran and is home to more than 210,000 farmers (44). So, large numbers of the population of the villages are farmers in this province, and the economy of most of the villagers is based on agriculture. Farmers in this province, like other farmers, have suffered a lot from COVID-19, so this province was studied as a community.

On the other hand, the conducted studies have not directly investigated the vulnerability of farmers in the face of COVID-19, so this study seeks to investigate the vulnerability of farmers to COVID-19 and categorize the mentioned vulnerability in order to carry out more consistent planning.

In other words, the research helps identify the dimensions of farmers' vulnerability to COVID-19 whose investigation can be effective in planning and policymaking for the removal of barriers and challenges and help enhance farmers' activities at critical times because the measurement of vulnerability is the starting point and prerequisite for crisis management during natural disasters and disease outbreaks.

## Methodology

This study is applied in terms of its goal and is descriptive-correlation in terms of data collection, monitoring, and variable control. The statistical population was composed of farmers in Kermanshah province (*N* = 126,900) in the west of Iran. At first, the province was divided into five parts (north, south, east, west, and center) according to geographical directions. Then, in each direction, the cities in question that were leading in terms of agriculture in the province were selected. The sample was taken by the multi-step stratified sampling technique with proportional allocation. The strata included the counties of Kermanshah province (Table 1). The sample size was determined to be 382 people using Krejcie and Morgan's table (45). The sample size for each county was specified by proportional allocation. Data were collected with a questionnaire, which was composed of two sections—one for demographic information and the other for the components of farmers' vulnerability to COVID-19. The components of the farmers' vulnerability were measured on a five-point Likert scale. The face and content validity of the research instrument was confirmed by a panel of experts. To confirm its reliability, 30 questionnaires were filled out by farmers outside the statistical sample in a pilot study to estimate Cronbach's alpha (α = 0.79). The collected data were analyzed by using the SPSSwin_23_ and AMOS_26_ software packages.

**Table 1:** The statistical population and sample sizes of different counties.

**Sr. No**.	**County**	**Statistical population**	**Sample size**
1	Eslamabad-e-Gharb	12,760	39
2	Paveh	30	1
3	Salas-e Babajani	3,307	10
4	Javanrud	2,033	7
5	Dalahu	6,707	22
6	Ravansar	9,554	30
7	Sonqor-va-Kolyai	15,440	46
8	Sarpol-e Zahab	10,021	30
9	Sahneh	12,144	37
10	Kermanshah	35,230	101
11	Kangavar	4,418	13
12	Gilanegharb	6,667	20
13	Qasreshirin	3,434	10
14	Harsin	5,155	16
Total		126,900	382

## Results

### Farmers' demographics

Farmers' demographic data revealed that 85.3 percent were male and 14.7 percent were female. Also, 78.4 percent were married and 21.6 percent were single. The farmers were, on average, 40.34 years old with a standard deviation of 11.98 and had, on average, 18.45 years of farming history with a standard deviation of 12.22. In terms of educational level, 17.3 percent had elementary school certificates, 39.5 percent had intermediate school certificates, 23.7 percent had diplomas, and 19.5 percent had academic education. On average, 59.7 percent of the studied farmers had no medical insurance. In terms of the product, 48.2 percent produced agronomic crops, 3.4 percent produced horticultural crops, 17.6 percent produced agronomic crops and animal products, 10.8 percent produced agronomic-horticultural crops, 14.5 percent produced animal products, and 5.5 percent produced seasonal crops. The average cultivated area of the farmers was 5.3 ha. The studied people were busy with farm activities for an average of 7.06 h/day.

### COVID-19 among farmers

The results showed that 47.3 percent of the studied farmers had contracted COVID-19. Also, 66.3 percent were aware of the infection symptoms and 62.1 percent were aware of how to prevent it. The respondents stated that their main source of information on COVID-19 was relatives (35.2%), social networks (31.1%), TV (27.9%), and radio (5.8%). Social distancing is reportedly taken care of at a moderate level (42.4%).

### Classification of farmers' vulnerability to COVID-19

First, we carried out exploratory factor analysis with varimax rotation on the data to derive a model in order to check whether the farmers' vulnerability to COVID-19 would replicate the four-factor structure within the studied population. Then, confirmatory factor analysis was employed to check the data structure, determine the number of dimensions of the variables, and validate the model (which is so far considered a predetermined model). To conduct exploratory factor analysis, the adequacy of the sample was checked by the Kaiser-Meyer-Olkin (KMO) test and the correlation of the variables was calculated by Bartlett's test of sphericity.

The KMO value was estimated at 0.871, implying the adequacy of the sample. Also, Bartlett's test of sphericity was found to be 4,234.534, which is significant at the *p* < 0.01 level and shows that the correlation of the data was not zero in the population. This analysis yielded four factors with eigenvalues of >1 and factor loadings of >0.5 using the varimax rotation. **Table 6** presents the eigenvalues, the percentage of variance accounted for, and the cumulative percentage of variance for each factor. As is observed, these four factors together captured 78.084 percent of the total variance. The variances captured by economic, agronomic-vulnerability, psychological-social, and environmental vulnerability were 41.170, 15.41, 9.706, and 3.798%, respectively. The results reflect the fact that the economic factor accounts for the greatest and the environmental factor accounts for the smallest fraction of vulnerability (Table 2).

**Table 2:** The exploratory factor analysis of farmers' vulnerability to COVID-19.

**Factor**	**Eigenvalue**	**Variance accounted for (%)**	**Cumulative variance (%)**
Economic vulnerability	14.259	49.170	49.170
Agronomic-vocational vulnerability	4.469	15.41	64.58
Psychological-social vulnerability	2.815	9.706	74.286
Environmental vulnerability	1.102	3.798	78.084

After the items were subjected to factor analysis, those whose factor loadings were >0.50 were retained. Table 3 presents the loadings of the items on the four factors. Accordingly, six items were loaded on the first factor (economic), five items on the second factor (agronomic-vocational), three items on the third factor (psychological-social), and three items on the fourth factor (environmental). Therefore, the economic factor with six items, the agronomic-vocational factor with five items, the psychological-social factor with three items, and the environmental factor with three items were not changed. So, the factor analysis was conducted, and the repetition of the four factors on the items showed the proper validity of the model derived from the exploratory factor analysis (the determined model).

**Table 3:** The factor loadings of the questionnaire items with the varimax rotation.

**Factor loadings**			
**Economic factor**	**Agronomic-vocational factor**	**Psychological-social factor**	**Environmental factor**
0.997	0.893		
0.823	0.893		
0.823	0.884	0.624	
0.788	0.850	0.676	0.683
0.590	0.707	0.824	0.686
0.536			0.839

### Status of farmers' vulnerability to COVID-19

The items constituting farmers' vulnerability to COVID-19 were ranked by the coefficient of variations (CV). The results about the economic vulnerability reveal that the highest rank is related to “the increase in production costs due to COVID-19” (CV = 0.278) and the lowest to “the decrease in providing the farmers with support services by the government” (CV = 0.55). Regarding psychological-social vulnerability, it is found that the highest rank is for “the increase in permanent or seasonal unemployment” (CV = 0.30) and the lowest for “the aggravation of criminal acts and insecurity in villages (addition, theft, etc.)” (CV = 0.42). The highest and lowest ranks in the agronomic-vocational vulnerability are related to “the decreased access to crop sale markets” (CV = 0.280) and “the decreased access to agricultural implements and machinery” (CV = 0.32), respectively. The results for the environmental vulnerability indicate that “the decreased control in pest and disease management at farms” (CV = 0.37) is at the top and “the conversion of arable lands and pastures to barren areas in rural areas” (CV = 0.48) is at the bottom of the list. Other findings are presented in Table 4.

**Table 4:** The ranking of the items constituting farmers' vulnerability to COVID-19.

**Vulnerabilities**		Mean^*^	**SD**	**CV**	**Rank**
Economic vulnerability	The increase in production costs	3.86	1.054	0.273	1
	The decline in purchasing power	3.92	1.09	0.278	2
	The increase in burrowing money from acquaintances	3.81	1.10	0.28	3
	The loss of financial capacity to repay loans	3.79	1.13	0.29	4
	The decrease in the sale prices of the products	3.60	1.11	0.30	5
	The decline in farm revenue	3.63	1.30	0.35	6
	The decrease in providing the farmers with support services by the government	2.54	1.40	0.55	7
Psychological-social vulnerability	Permanent or seasonal unemployment	3.61	1.11	0.30	1
	Farmers' more dependence on the government	3.37	1.152	0.34	2
	Career change from farming to other jobs	3.23	1.156	0.35	3
	Dissatisfaction with governmental agencies	3.34	1.28	0.38	4
	The decrease in people's participation and cooperation in village affairs	2.93	1.21	0.41	5
	The aggravation of criminal acts and insecurity in villages (addition, theft, etc.)	2.93	1.25	0.42	6
Agronomic-vocational vulnerability	The decreased access to crop sale markets	3.78	1.07	0.28	1
	The decreased access to labor for agricultural operations	3.84	1.14	0.29	2
	Changes in cropping pattern	3.57	1.10	0.30	3
	The decreased access to agricultural inputs	3.64	1.20	0.32	4
	The decline in crop yields	3.35	1.190	0.35	5
	The decreased access to agricultural implements and machinery	3.31	1.26	0.38	6
Environmental vulnerability	Farmers' decreased control in pest and disease management at farms	3.36	1.25	0.37	1
	The reduction of vegetation and pasture cover in the rural area	3.44	1.31	0.38	2
	The reduction of attention to the environmental health of the rural area	3.11	1.33	0.42	3
	The conversion of arable lands and pastures to barren areas in rural areas	2.85	1.25	0.48	4

* Range of means: 1, very low; 2, low; 3, moderate; 4, high; 5, very high.

The mean dimensions of the farmers' vulnerability to COVID-19 were compared by a one-sample *t*-test. The results revealed significant differences among the economic, psychological-social, agronomic-vocational, and environmental dimensions at the *p* < 0.01 level with mean intervals. Based on the results of the one-sample *t*-test, the upper and lower bounds of the psychological-social and agronomic-vocational vulnerability dimensions were negative, implying that the mean of the population was significantly smaller than the mean interval of the variables. In other words, the means of psychological-social and agronomic-vocational vulnerabilities to COVID-19 were lower than the moderate level. Other findings are presented in Table 5.

**Table 5:** The comparison of mean dimensions of farmers' vulnerability with the mean intervals.

**Dimensions**	**Test value**	**Mean**	**Sig**.	**Mean difference**	***t***	**95% confidence interval of the difference**	
						**Lower**	**Upper**
Economic vulnerability	21	26.32	0.000	5.32303	17.115	4.7115	5.9345
Psychological-social vulnerability	18	14.20	0.000	−3.79737	−19.805	−4.1744	−3.4204
Agronomic-vocational vulnerability	18	20.16	0.000	2.16316	12.667	−0.3178	0.6441
Environmental vulnerability	12	12.77	0.000	0.77895	−187.878	0.4103	2.1476

Friedman's test was used to compare the means of the variables of economic, psychological-social, agronomic-vocational, and environmental vulnerability within the studied sample. The results showed significant differences at the 0.001 level among the means of the studied vulnerability dimensions, so the participants believe that the dimensions of vulnerability are not equally important. The highest mean was assigned to the variable of agronomic-vocational vulnerability (2.86) and the lowest to the variable of psychological-social vulnerability (1.75). Therefore, an implication of the epidemic is the greater vulnerability in agronomic-vocational factors among farmers (Table 6).

**Table 6:** The comparison of the means of the vulnerability dimensions.

**Vulnerability dimension**	**Mean**	**Mean rank**	**Chi-square**	**df**	**Asymp. Sig**.
Economic vulnerability	26.32	3.94	876.898	3	0.000
Psychological-social vulnerability	14.24	1.75			
Agronomic-vocational vulnerability	16.18	2.86			
Environmental vulnerability	12.79	1.46			

Convergent validity is a quantitative measure that shows the degree of internal correlation and alignment of the items of measurement of a category, so in this study, convergent validity was investigated by two criteria:

- Average Variance Extracted or AVE (46).
- Composite Reliability or CR (47).

Since the AVE criterion indicates the average variance shared between each construct with its own indicators and shows the correlation between a construct and its indicators, the higher it is, the more fit the construct is. Accordingly, convergent validity exists when AVE is >0.5 and CR of 0.7 and also the CR value should be larger than AVE. In this regard, the results showed that there was convergent validity in the variables of this study because the convergent narrative conditions that CR > 0.7 CR > AVE and AVE > 0.5 are established in the model variables (Table 7).

**Table 7:** The measurement coefficients, significance levels of the confirmatory factor analysis, and the validity and reliability of the variables.

**Latent variables**	**Observed variables**	**Standardized loading**	**AVE**	**CR**	***T*-value**
Economic	The increase in burrowing money from acquaintances	0.86	0.57	0.88	11.17
	The increase in production costs	0.91			11.76
	The decrease in the sale prices of the products	0.56			4.76
	The loss of financial capacity to repay loans	0.66			6.68
	The decline in purchasing power	0.90			12.53
	The decline in farm revenue	0.53			4.59
Psychological-social	Career change from farming to other jobs	0.64	0.50	0.75	4.94
	Farmers' more dependence on the government	0.67			5.1
	Permanent or seasonal unemployment	0.81			8.08
Environmental	Farmers' decreased control in pest and disease management at farms	0.81	0.50	0.74	9.87
	The reduction of vegetation and pasture cover in the rural area	0.69			5.56
	The reduction of attention to the environmental health of the rural area	0.61			5.13
Agronomic-vocational	The decline in crop yields	0.57	0.54	0.85	4.38
	The decreased access to crop sale markets	0.84			10.11
	The decreased access to agricultural inputs	0.65			6.45
	Changes in cropping pattern	0.77			8.02
	The decreased access to labor for agricultural operations	0.82			8.23

**Figure 1:**
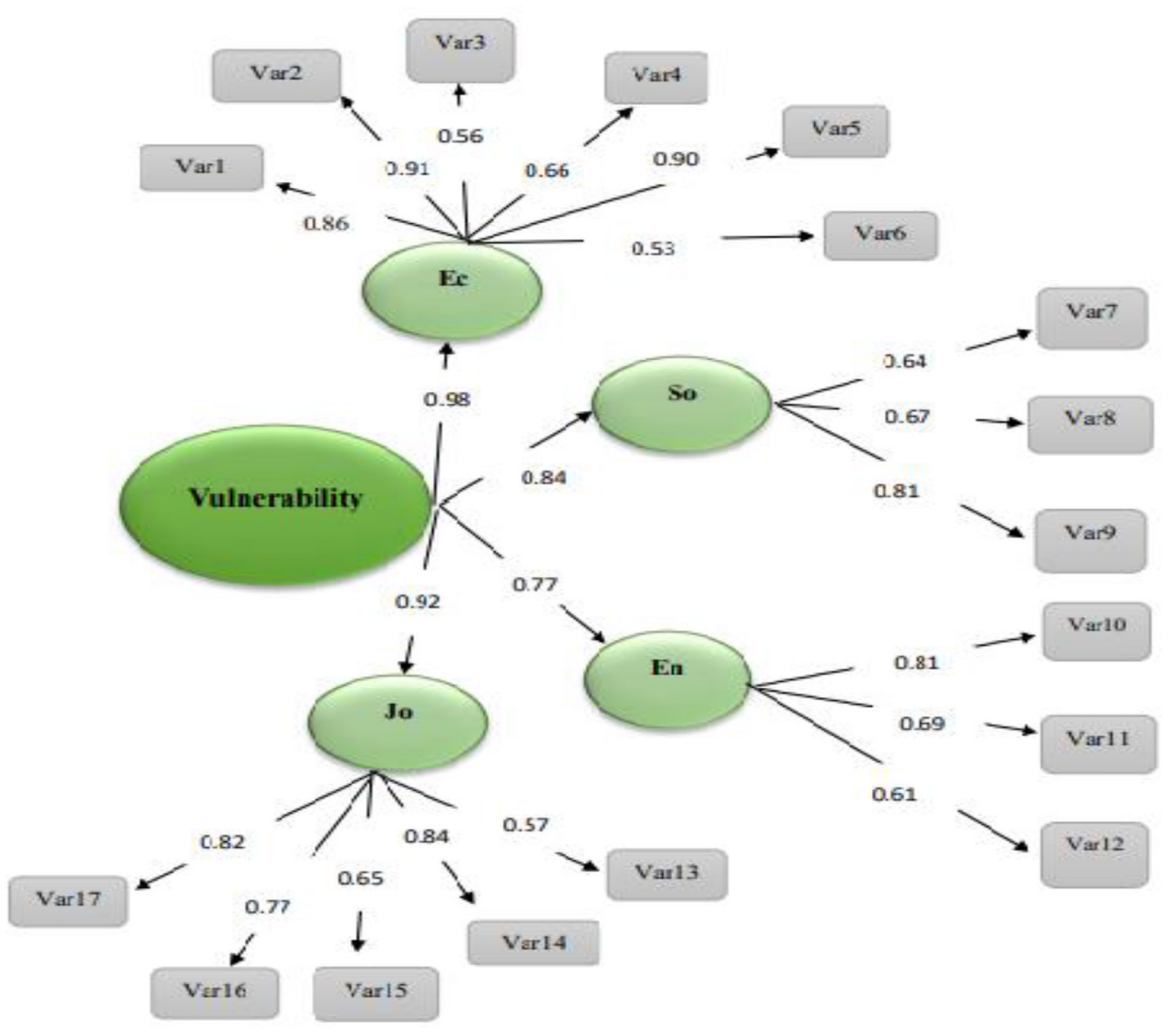
The standardized coefficients of the paths in the four-factor structure of farmers' vulnerability to COVID-19.

The consistency of the items included in the research model in terms of content and fundamental dimensions was validated by confirmatory factor analysis. There are several fitness indicators for the assessment of confirmatory factor models. The present research employed chi-square per degree of freedom (χ2/df), root mean square residual (RMR), goodness-of-fit index (GFI), and adjusted goodness-of-fit index (AGFI). The chi-square tests the hypothesis that the target model is consistent with the covariance pattern among the observed variables. The lower values of χ2/df show the higher fitness of the model. RMR means the difference between the elements of the matrix observed in the sample group and the elements of the estimated or predicted matrices assuming that the target model is valid. Figure 1 depicts the standardized coefficients of the paths in the four-factor structure of farmers' vulnerability to COVID-19.

In Figure 1, Ec stands for the economic factor, So stands for the psychological-social factor, En stands for the environmental factor, Jo stands for the agronomic-vocational factor, and Var stands for the variable or items. Table 8 summarizes the fitness indicators of the model. The results reveal that all indicators are at acceptable levels, so the data properly fit the four-factor structure of the model and the items are consistent with the underlying structure. Therefore, the four-factor model fits well.

**Table 8:** The goodness-of-fit indicators for the model derived from farmers' vulnerability to COVID-19.

**Test**	Recommended value^*^	**Estimated value**
Adjusted goodness of fit index	AGFI > 0.9	0.90
Normed chi-square (χ^2^/df)	x2/df^**^ < 5	2.11
Root mean squared error of approximation	RMSEA < 0.08	0.06
Root mean square residual	RMR < 0.9	0.54
Comparative fit index	CFI > 0.90	0.92
Goodness fit index	GFI > 0.9	0.93

* Byrne (48);

** df: 265.

## Discussion and conclusions

The study of vulnerability is the first step, the starting point, and a prerequisite for crisis management, and plans for alleviating crisis effects are necessary for reducing the vulnerability of rural communities. Since the COVID-19 pandemic has had adverse impacts on production sectors including agriculture with deep negative effects on this sector due to the huge disruptions made in the supply chain, which has severely threatened farmers as the group of people who are actively involved in food production and has imposed them heavy financial losses and destructive psychological damages (49), the present research aimed to help understand farmers' vulnerability to COVID-19. The findings showed that the studied farmers receive the most information from their relatives. Therefore, extension agents and other trainers should provide the necessary training on crisis management to experts and local leaders and use the position of local leaders to disseminate information among other local people (50). More than half of the people studied had elementary school or intermediate school certificates, so in the design of educational programs attention should be paid to the simplicity of the content and the use of appropriate educational methods. In relation to the fact that farmers have incurred economic damage due to the COVID-19 virus and considering that the main occupation of rural people is agriculture, in order to prevent farmers from suffering it is necessary to provide technical and professional training and develop the skills of farmers to empower them. Farmers can use their skills and capabilities in the face of similar crises.

Based on the results, the vulnerability of the farmers in Kermanshah province to COVID-19 was significant in all dimensions including economic, psychological-social, environmental, and agronomic-vocational dimensions. The agronomic-vocational factor was found to be the most important factor responsible for the damages of the COVID-19 pandemic. Among the items related to this factor, the effects of the pandemic on the reduction of access to crop sales markets and the reduction of access to labor for farm activities were the most important ones. In this regard, Lopez-Ridaura et al. also emphasize that restrictions result in labor shortages for crop harvest, making it difficult for farmers to supply their crops to the market (51). It is inferred that the pandemic has influenced farmers' activities from various aspects. Regarding the agronomic-vocational activities in the agricultural sector, farmers need support to be able to produce the required food and raw materials for accomplishing predetermined goals and minimizing the need for imports. Otherwise, society should spend the exchange required for industrial development on the importation of food and agricultural commodities, which will surely impair economic growth (52). Furthermore, extension agents can teach farmers how to market and sell their products in the virtual world. As such, farmers and rural producers will be motivated to sustain their activities during crises.

Economic vulnerability was ranked the second most important dimension of vulnerability to COVID-19. According to the results, the reduction of purchasing power, the increase in production costs, and the increase in money borrowed from friends and relatives were the most important effects of the pandemic on the farmers. It can be said that the increasing economic pressure on farmers due to the pandemic is evident in various economic aspects. In these conditions, the government can help farmers and reduce the financial pressure and therefore mental pressure on them by providing support packages, providing easy-to-take facilities, reducing taxes or exempting them from some taxes, adjusting the prices of energy carriers, and making plans for the guaranteed purchase of their crops. FAO confirmed the economic burden on farmers during the COVID-19 pandemic (39). Phillipson et al. (53) conclude that COVID-19 has had significant impacts on the rural economy (51). In this regard, Ahmadyan notes that suitable policies are necessary for reinforcing investment in different economic sectors. This requires stability in national monetary and financial policies. Policymakers are recommended to consider support-credit policies, e.g., the supply of low-interest loans with a long or delayed repayment period (52). Samkhaniani emphasized the economic pressure of COVID-19 on farmers. Accordingly, restrictions in the delivery of agricultural commodities from the production point to final consumers have reduced farmers' revenue significantly (54).

In the ranking of the dimension of vulnerability to COVID-19, environmental vulnerability was ranked third. In this dimension, the effects of the pandemic on reducing farmers' control over pest and disease management at farms and the reduction of vegetation and pasture cover were the most important environmental damages according to the farmers. In this regard, the Office of Agricultural Extension and Education should focus on education in this field still more. Iese et al. also emphasized the environmental damages of the COVID-19 pandemic (41).

Regarding the psychological-social vulnerability, permanent or seasonal unemployment and farmers' dependence on the government were the most important damages of COVID-19. In this regard, it can be inferred that restrictions, shutdowns, and fear of susceptibility to infection caused unemployment among farmers. Yao et al. addressed the mental damages and effects of COVID-19 and stated that shutdown and self-isolation can most probably impact people's mental health adversely. Isolation from relatives, the loss of freedom, boredom, and unreliability of the conditions can aggravate people's mental health (55). In this respect, the efforts of farmers-related organizations to train them in crisis management can partially be effective in reducing the pressure on them because rural areas, especially in developing countries, are less prepared to cope with the direct and indirect effects of this crisis (56). This has been corroborated by Moradhaseli et al. that this pandemic affects farmers and their families, including the increased likelihood of suicide and post-traumatic stress disorder (57).

The results show that these factors have inflicted damage on farmers in different fields. Accordingly, the status of the farmers' vulnerability was developed in the research population for the measurement of the economic, agronomic-vocational, psychological-social, and environmental structures. Based on the results of the confirmatory factor analysis, despite extensive damages, vulnerability is fiercer in four factors (economic, agronomic-vocational, psychological-social, and environmental). So, this research has some theoretical achievements. Firstly, the research portrayed the multi-factor nature of farmers' vulnerability to COVID-19. In addition, these factors lend themselves to assessment. Above all, a model of the correlation of these dimensions was derived. In total, the study of vulnerability at different levels allows a deeper understanding of vulnerability processes in the conditions faced by farmers.

In general, the COVID-19 pandemic is one of the most universal phenomena in human history whose dimensions have influenced all people on the earth and all human actions and activities in various facets and aspects. Farmers have not only been no exception, but they have also been among the groups that have been most deeply influenced by the pandemic. In this regard, organizations and institutions in charge of different agricultural sections should focus their efforts and activities on developing and improving alternative and new management systems in the agricultural sector and designing and implementing targeted and effective training for farmers to deal with critical conditions. In general, one of the policies that can be adopted according to the results of this research is to help farmers in the direction of risk management. Risk management can be done through risk transfer (the most common methods of risk transfer in agriculture are the use of forward markets, pre-sale contracts, guaranteed prices, and insurance), training on how to deal with risk, and using the experiences of farmers. Another proposed policy is to help strengthen the livelihood of farmers by providing a suitable platform for providing banking facilities, non-governmental support funds, the ability to market products and the prosperity of domestic markets, income stability, and job stability. Another policy is to help solve farmers' agricultural problems by raising their awareness and helping to monitor and prevent pests. Finally, the proposed policy is in line with social-psychological issues through conducting field research activities (to identify the attitude and perception of people regarding the crisis) and creating a specialized working group at the village level.

## Data availability statement

The original contributions presented in the study are included in the article/Supplementary material, further inquiries can be directed to the corresponding author/s.

## Author contributions

SM involved in survey development and data management. PA and SH conducted conceptualization and statistical analyses. HK provided substantive feedback and contributed to the drafting of the manuscript. All authors contributed to the article and approved the submitted version.
