# Supplementary material for: Typology of Iranian farmers' vulnerability to the COVID-19 outbreak
Source: Front Public Health. 2022 Dec 22;10:1018406. doi: 10.3389/fpubh.2022.1018406 (PMC9814122; doi:10.3389/fpubh.2022.1018406)
Supplement: Supplementary file 1 [file Data_Sheet_1.PDF]

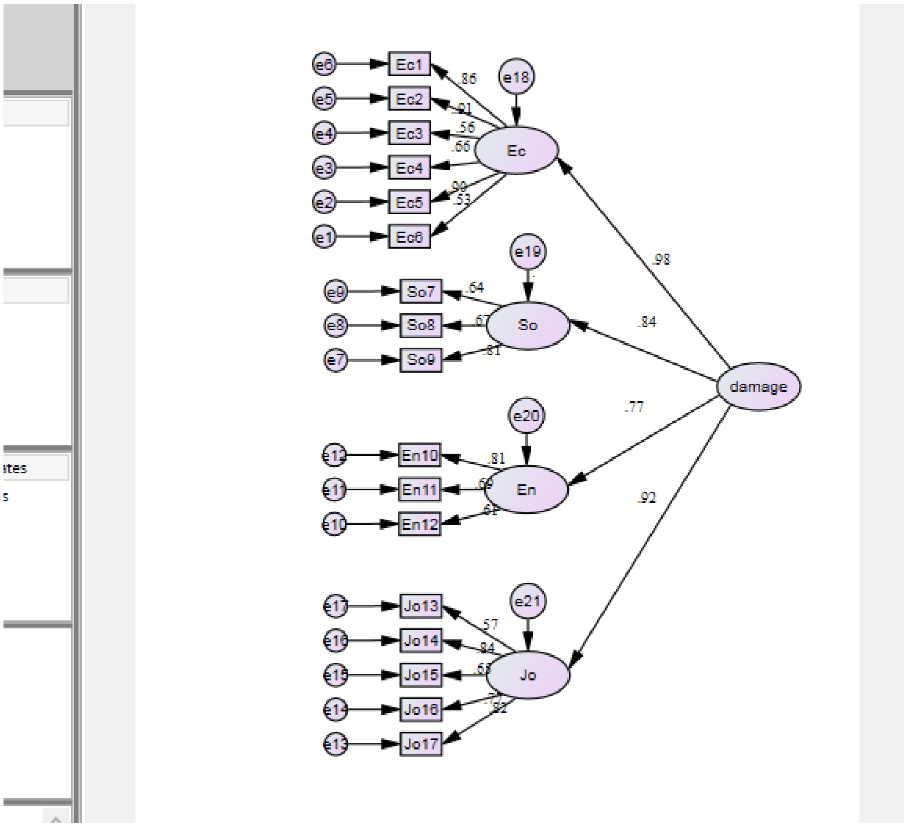

Amos Output

3 7 0

- ta1.amw
  - Analysis Summary
  - Notes for Group
  - Variable Summary
  - Parameter Summary
  - Assessment of normality
  - Observations farthest from the centroid (Mahalanobis distance)
  - Notes for Model
  - Estimates
  - Notes for Group/Model
  - Minimization History
  - Pairwise Parameter Comparisons
  - Model Fit
  - Execution Time

Group number 1

Default model

Model Fit Summary

CMIN

Model	NPAR	CMIN	DF	P	CMIN/DF
Default model	93	2098.816	265	.000	2.114
Saturated model	353	.000	0		
Independence model	40	3293.799	288	.000	6.866

RMR, GFI

Model	RMR	GFI	AGFI	PGFI
Default model	.545	.938	.906	.882
Saturated model	.000	1.000		
Independence model	.806	.895	.879	.894

Baseline Comparisons

Model	NFI Delta1	RFI rho1	IFI Delta2	TLI rho2	CFI
Default model	.882	.716	.928	.862	.921
Saturated model	1.000		1.000		1.000
Independence model	.000	.000	.000	.000	.000

Parsimony-Adjusted Measures

Model	PRATIO	PNFI	PCFI
Default model	.931	.833	.851
Saturated model	.000	.000	.000
Independence model	1.000	.000	.000

Amos Output

3 7 0

- ta1.amw
  - Analysis Summary
  - Notes for Group
  - Variable Summary
  - Parameter Summary
  - Assessment of normality
  - Observations farthest from the centroid (Mahalanobis distance)
  - Notes for Model
  - Estimates
  - Notes for Group/Model
  - Minimization History
  - Pairwise Parameter Comparisons
  - Model Fit
  - Execution Time

NCP

Model	NCP	LO 90	HI 90
Default model	1185.816	1083.291	2095.783
Saturated model	.000	.000	.000
Independence model	3157.799	3006.228	3316.736

FMIN

Model	FMIN	F0	LO 90	HI 90
Default model	2.081	1.867	1.673	2.075
Saturated model	.000	.000	.000	.000
Independence model	4.764	4.987	3.890	4.698

RMSEA

Model	RMSEA	LO 90	HI 90	PCLOSE
Default model	.068	.061	.073	.000
Independence model	.073	.067	.080	.000

AIC

Amos Output

3 7 0

- ta1.amw
  - Analysis Summary
  - Notes for Group
  - Variable Summary
  - Parameter Summary
  - Assessment of normality
  - Observations farthest from the centroid (Mahalanobis distance)
  - Notes for Model
  - Estimates
  - Notes for Group/Model
  - Minimization History
  - Pairwise Parameter Comparisons
  - Model Fit
  - Execution Time

AIC

Model	AIC	BCC	BIC	CAIC
Default model	287.901	291.640	349.656	389.656
Saturated model	314.000	356.903	459.477	212.467
Independence model	337.968	367.893	412.421	425.438

ECVI

Model	ECVI	LO 90	HI 90	MECVI
Default model	.694	.732	.612	.876
Saturated model	1.58	.580	.580	.600
Independence model	7.09	4.552	4.798	4.531

HOELTER

Model	HOELTER .05	HOELTER .01
Default model	18	24
Independence model	6	10

Amos Output

3 7 0

- ta1.amw
  - Analysis Summary
  - Notes for Group
  - Variable Summary
  - Parameter Summary
  - Assessment of normality
  - Observations farthest from the centroid (Mahalanobis distance)
  - Notes for Model
  - Estimates
  - Notes for Group/Model
  - Minimization History
  - Pairwise Parameter Comparisons
  - Model Fit
  - Execution Time

Group number 1

Estimates (Group number 1 - Default model)

Scalar Estimates (Group number 1 - Default model)

Maximum Likelihood Estimates

Regression Weights: (Group number 1 - Default model)

			Estimate	S.E.	C.R.	P	Label
e6oa1	<---	Ec	1.000				
e5oa1	<---	Ec	.889	.054	6.891	***	par_1
e4oa1	<---	Ec	1.603	.335	4.778	***	par_2
e3oa1	<---	Ec	2.391	.464	5.150	***	par_3
e2oa1	<---	Ec	1.762	.353	4.997	***	par_4
e1oa1	<---	Ec	3.643	.477	5.625	***	par_5
s3ea1	<---	So	1.000				
s2ea1	<---	So	23.446	4.503	8.556	***	par_6
s1ea1	<---	So	1.372	.180	7.637	***	par_7
e11oa1	<---	En	1.000				
e10oa1	<---	En	.250	.026	10.387	***	par_8
e9oa1	<---	En	4.824	1.443	3.667	***	par_9
j5oa1	<---	Jo	1.000				
j4oa1	<---	Jo	6.032	1.503	4.001	***	par_10
j3ooa1	<---	Jo	2.488	.572	4.349	***	par_11
j2oa1	<---	Jo	6.543	1.913	8.103	***	par_12
j1oa1	<---	Jo	4.823	1.410	7.340	***	par_13

Amos Output

3 | 7 | 0

- ta1.amw
  - Analysis Summary
  - Notes for Group
  - Variable Summary
  - Parameter Summary
  - Assessment of normality
  - Observations farthest from the centroid (Mahalanobis distance)
  - Notes for Model
  - Estimates
  - Notes for Group/Model
  - Minimization History
  - Pairwise Parameter Comparisons
  - Model Fit
  - Execution Time

Standardized Regression Weights: (Group number 1 - Default model)

			Estimate
e6oa1	<---	Ec	.532
e5oa1	<---	Ec	.901
e4oa1	<---	Ec	.660
e3oa1	<---	Ec	.561
e2oa1	<---	Ec	.926
e1oa1	<---	Ec	.860
s3ea1	<---	So	.811
s2ea1	<---	So	.672
s1ea1	<---	So	.639
e11oa1	<---	En	.614
e10oa1	<---	En	.691
e9oa1	<---	En	.809
j5oa1	<---	Jo	.818
j4oa1	<---	Jo	.770
j3ooa1	<---	Jo	.653
j2oa1	<---	Jo	.836
j1oa1	<---	Jo	.567

Amos Output

3 | 7 | 0

- ta1.amw
  - Analysis Summary
  - Notes for Group
  - Variable Summary
  - Parameter Summary
  - Assessment of normality
  - Observations farthest from the centroid (Mahalanobis distance)
  - Notes for Model
  - Estimates
  - Notes for Group/Model
  - Minimization History
  - Pairwise Parameter Comparisons
  - Model Fit
  - Execution Time

Group number 1

Variances: (Group number 1 - Default model)

	Estimate	S.E.	C.R.	P	Label
Ec	.347	.127	2.724	***	par_20
So	.498	.102	4.888	***	par_21
En	4.161	.073	5.178	***	par_22
Jo	.037	.318	12.090	***	par_23
e1	4.281	.272	15.742	***	par_24
e2	6.422	.395	16.245	***	par_25
e3	3.536	.242	14.633	***	par_26
e4	2.916	.254	11.504	***	par_27
e5	2.676	.197	13.597	***	par_28
e6	1.985	.122	16.232	***	par_29
e7	1.337	.106	12.653	***	par_30
e8	1.710	.105	16.221	***	par_31
e9	1.675	.162	10.355	***	par_32
e10	4.470	.242	.876	***	par_33
e11	7.939	.099	7.568	***	par_34
e12	6.452	.403	16.019	***	par_35
e13	1.032	.064	16.215	***	par_36
e14	2.693	.166	16.260	***	par_37
e15	1.411	.098	14.420	***	par_38
e16	1.052	.065	16.244	***	par_39
e17	.424	.485	.874	***	par_40

Amos Output

3 | 7 | 0

- ta1.amw
  - Analysis Summary
  - Notes for Group
  - Variable Summary
  - Parameter Summary
  - Assessment of normality
  - Observations farthest from the centroid (Mahalanobis distance)
  - Notes for Model
  - Estimates
  - Notes for Group/Model
  - Minimization History
  - Pairwise Parameter Comparisons
  - Model Fit
  - Execution Time

Indirect Effects (Group number 1 - Default model)

	Jo	En	So	Ec
j1oa1	.000	.000	.000	.000
j2oa1	.000	.000	.000	.000
j3ooa1	.000	.000	.000	.000
j4oa1	.000	.000	.000	.000
j5oa1	.000	.000	.000	.000
e9oa1	.000	.000	.000	.000
e10oa1	.000	.000	.000	.000
e11oa1	.000	.000	.000	.000
s1ea1	.000	.000	.000	.000
s2ea1	.000	.000	.000	.000
s3ea1	.000	.000	.000	.000
e1oa1	.000	.000	.000	.000
e2oa1	.000	.000	.000	.000
e3oa1	.000	.000	.000	.000
e4oa1	.000	.000	.000	.000
e5oa1	.000	.000	.000	.000
e6oa1	.000	.000	.000	.000

Standardized Indirect Effects (Group number 1 - Default model)

Amos Output

- ta1.amw
  - Analysis Summary
  - Notes for Group
  - Variable Summary
  - Parameter Summary
  - Assessment of normality
  - Observations farthest from the centroid (Mahalanobis distance)
  - Notes for Model
  - Estimates
  - Notes for Group/Model
  - Minimization History
  - Pairwise Parameter Comparisons
  - Model Fit
  - Execution Time

Standardized Indirect Effects (Group number 1 - Default model)

	Jo	En	So	Ec
j1oa1	.000	.000	.000	.000
j2oa1	.000	.000	.000	.000
j3ooa1	.000	.000	.000	.000
j4oa1	.000	.000	.000	.000
j5oa1	.000	.000	.000	.000
e9oa1	.000	.000	.000	.000
e10oa1	.000	.000	.000	.000
e11oa1	.000	.000	.000	.000
s1ea1	.000	.000	.000	.000
s2ea1	.000	.000	.000	.000
s3ea1	.000	.000	.000	.000
e1oa1	.000	.000	.000	.000
e2oa1	.000	.000	.000	.000
e3oa1	.000	.000	.000	.000
e4oa1	.000	.000	.000	.000
e5oa1	.000	.000	.000	.000
e6oa1	.000	.000	.000	.000
